# The size effect on mechanical and permeability properties of porous bone structures fabricated by selective laser melting

**DOI:** 10.3389/fbioe.2026.1846035

**Published:** 2026-07-31

**Authors:** Yongtao Lyu, Hongrui Cao, Peiqi Jing, Yunfei Lan, Sergei Bosiakov, Haytham Elgazzar, Guangyu Li, Yadong Liu

**Affiliations:** 1 Department of Spinal Surgery, Central Hospital of Dalian University of Technology, Dalian University of Technology, Dalian, China; 2 School of Mechanics and Aerospace Engineering, Dalian University of Technology, Dalian, China; 3 DUT-BSU Joint Institute, Dalian University of Technology, Dalian, China; 4 Faculty of Mechanics and Mathematics, Belarusian State University, Minsk, Belarus; 5 The Advanced Digital Manufacturing Department, Central Metallurgical Research and Development Institute, El-Tebbin, Helwan, Egypt; 6 School of Materials Science and Engineering, Dalian University of Technology, Dalian, China

**Keywords:** Bone scafolds, computational fluid dynamics, finite element analysis, porous structures, selective laser melting, size effect, triply periodic minimal surface

## Abstract

Porous structures are extensively utilized in bone tissue engineering for their lightweight nature, high strength-to-weight ratio, and superior energy absorption capacity. However, achieving optimal scaffold performance through precise dimension control remains a significant design challenge. This study systematically investigates the effect of unit-cell size on the mechanical and fluid transport properties of porous bone structures, aiming to provide guidance for scaffold design. Various porous structures with controlled unit cell sizes were fabricated via Selective Laser Melting (SLM) and evaluated under compressive loading. A complementary finite element (FE) model was developed to simulate mechanical responses and extract equivalent elastic modulus and compressive strength, while computational fluid dynamics (CFD) simulations were conducted on 4 × 4 × 4 arrays to calculate permeability. Results demonstrate that the Gyroid unit cell exhibited the most uniform stress distribution among the three studied architectures (Cube, Octa, Gyroid), and reducing unit cell size effectively mitigated stress concentrations to enhance structural stability. At a constant porosity, the compressive strength of uniform porous structures decreased with increasing unit-cell size, whereas the elastic modulus showed relatively low sensitivity to size variation. Meanwhile, permeability increased markedly with increasing unit-cell size. This study confirms that unit cell size profoundly impacts both the mechanical integrity and permeability of porous bone structures: smaller unit cells are recommended for applications prioritizing high compressive strength, whereas larger unit cells are more favorable for enhanced fluid transport.

## 1 Introduction

Porous structures are characterized by a range of advantageous properties, including low density, high specific surface area, effective thermal insulation, and notable vibration damping capacity (Zhang et al., 2022; Kashimbetova et al., 2023; Zeng et al., 2023; Tüzemen et al., 2022; Liu et al., 2022; Sun et al., 2023). Consequently, these structures are increasingly employed in high-performance engineering applications, biomedical engineering, aerospace, and automotive components, especially in the aspect of bone scaffolds. Among various porous architectures, those based on Triply Periodic Minimal Surfaces (TPMS) have garnered significant research interest owing to their geometrically smooth and self-supporting nature and the precise control over their morphology enabled by additive manufacturing techniques (Vyavahare et al., 2023; Li et al., 2023a; Li et al., 2023b; Nasim and Galvanetto, 2021; Xu et al., 2023; Tran et al., 2023; Xiong et al., 2023).

The performance of porous structures is intrinsically linked to their unit cell dimensions, a phenomenon broadly categorised as the size effect (Alam et al., 2012; Alam et al., 2014; Morel, 2007). Recent investigations have established that the pore size, porosity, and pore shape collectively govern the macroscopic behaviour of these structures (Bukka and Sarin, 2024; Dube Kerme et al., 2024). This finding revealed the critical role of meticulously tuning the unit cell geometry during the design process. Therefore, selecting an appropriate unit cell size is paramount for the development of high-performance porous structures. This tailoring capability is particularly valuable in bone scaffolds, where components must simultaneously satisfy stringent requirements for low weight, high strength, and efficient permeability management.

Beyond the size, the fundamental geometry of the unit cell is a decisive factor influencing the overall performance. Research trends have progressively shifted from conventional strut-based lattices (e.g., Cube, Octahedron, hereafter referred to as Octa) to smoothly curved TPMS-based structures (e.g., Gyroid, Diamond) (Yan et al., 2012; Khaderi et al., 2014). Conventional lattices often feature sharp angles and junctions, which can act as stress concentrators and lead to nonuniform load distributions. In contrast, TPMS structures possess a zero mean curvature (Al-Ketan et al., 2020), promoting a smoother stress flow. Their high surface area-to-volume ratio also contributes to enhanced specific mechanical properties (Hu et al., 2022). Moreover, the implicit mathematical definition of TPMS structures enables precise control over parameters such as pore size and porosity, facilitating the fabrication of porous structures with tailored mechanical properties. This precise control makes the TPMS structures an ideal platform for systematically investigating the size effects.

In addition to regular lattice and TPMS-based porous structures, irregular porous structures generated by Voronoi or Laguerre–Voronoi tessellation algorithms have also attracted considerable attention, particularly for bone scaffold applications. Such irregular architectures may better mimic the stochastic morphology of trabecular bone and may allow the introduction of pore-size distribution and spatial heterogeneity. For example, LVT-based titanium scaffolds have been designed for tissue engineering applications, and their elastic properties have been investigated through both experimental compression tests and computational simulations (Cwieka et al., 2024). Compared with regular periodic porous structures, irregular LVT-based structures may involve coupled variations in local pore size, strut orientation, pore-volume distribution, and connectivity. These coupled geometric features can make it more challenging to isolate the independent contribution of a single design parameter. Compared with irregular porous structures, regular periodic structures may offer relatively straightforward control of porosity, unit-cell size, and unit-cell topology. This controllability is useful in the present study because the main objective was to examine the influence of unit-cell size while reducing the interference of porosity variation and stochastic geometric heterogeneity. Therefore, regular Cube, Octa, and Gyroid architectures were selected in this study as representative and controllable model systems, rather than as structures claimed to be generally superior to irregular porous architectures. To understand the influence of the size effects, numerous experimental and numerical studies have been conducted focusing on the unit cell type and porosity (Xu et al., 2019; Egan et al., 2017; Al-Ketan et al., 2018; Kladovasilakis et al., 2021; Jung and Buehler, 2018). For instance, comparative analyses of the mechanical properties of TPMS and conventional truss structures, as well as studies on cubic and octahedral cells, have been reported. Jung et al. modeled TPMS structures of Primitive (P), Diamond (D), and Gyroid (G) at different scales, confirming the combined effects of architecture and size on elastic modulus, strength, and fracture behavior. Their work highlighted that only the Gyroid structure exhibited isotropic elastic properties, providing valuable insights for structural optimization.

While prior research has significantly advanced our understanding of how the unit cell type affects the porous structure properties, a systematic investigation explicitly focusing on the isolated effect of the unit cell size remains relatively scarce. Unlike previous studies that mainly investigated the coupled effects of pore size, porosity, or unit-cell topology, the present work focuses on isolating the role of unit cell size under a constant target porosity. By fixing the porosity at 70% for all uniform porous structures, the influence of porosity variation was minimized, allowing the independent effect of unit cell size on mechanical response and permeability to be evaluated more directly. In addition, three representative unit-cell topologies, including Cube, Octa, and Gyroid, were compared under the same porosity and size conditions, and the analysis was further extended to functionally graded Gyroid structures. Therefore, this study provides a controlled structure–property comparison for understanding how unit cell size regulates both load-bearing capacity and fluid transport in porous bone scaffold design. The present work is not intended to propose a new porous topology, but rather to provide a controlled experimental–numerical comparison of unit-cell size effects under a fixed target porosity. By maintaining a constant porosity of 70% for all uniform porous structures, the possible influence of porosity variation was reduced, which may allow the role of unit-cell size in regulating mechanical response and permeability to be evaluated more directly. To address this gap, this study comprehensively investigates the influence of unit cell size on the mechanical properties and permeability of porous structures. Three types of uniform structures (Cube, Octa, Gyroid) and gradient Gyroid structures with varying unit-cell sizes were fabricated via SLM. Their compressive performance was evaluated through experiments and finite element analysis, and CFD simulations were used to assess their permeability. This integrated approach aims to establish clear structure-property-performance relationships and provide valuable design guidelines for high-performance, lightweight components in fields such as biomedical engineering, aerospace and automotive engineering, where balancing mechanical integrity with functional requirements, such as fluid flow, is critical.

## 2 Materials and methods

### 2.1 Design of porous structures

In this study, three types of unit cells were selected for investigation: the simple cubic (Cube) structure, octahedral (Octa) structure, and TPMS-based Gyroid structure (Feng et al., 2022). Both Cube and Octa structures are known for their favorable compressive properties, shear resistance, and permeability along the principal axes. Design simplicity and ease of manufacturing are offered by the Cube structure (Ma et al., 2022), whereas a high compressive modulus is typically exhibited by the Octa structure. These strut-based cells were designed by defining the radius of the internal tangent circle within their cross-sections to achieve a target porosity of 70%. Unit cell sizes of 1.5 mm, 2.5 mm, and 3.0 mm were constructed. The selection of these three unit cell sizes was based on both structural design considerations and potential scaffold requirements. The 2.5 mm unit cell size was selected as a commonly used reference size in porous scaffold design, while the 1.5 mm size was used to examine the influence of smaller unit cells on stress distribution and flow resistance. The 3.0 mm size was included as a larger reference to evaluate the effect of enlarged flow channels. From a bone scaffold perspective, reducing the apparent elastic modulus of metallic implants is important for mitigating stress shielding, while interconnected pores are required to provide space for tissue ingrowth and channels for fluid transport. Therefore, this size range was selected to evaluate the trade-off between mechanical compatibility and permeability under a constant target porosity. The corresponding radii for the Cube and Octa structures are listed in Table 1.

**TABLE 1:** The radius *r* of internal tangent circles for different unit cell sizes.

Size (mm)	1.5	2.5	3.0
Cube	0.27	0.45	0.54
Octa	0.19	0.31	0.37

All strut radii were calculated to achieve a constant target porosity of 70% for all uniform porous structures.

A minimal surface is defined as a surface whose area is locally minimized. TPMS structures have the defining property of zero mean curvature at every point. The morphology of a TPMS is governed by an implicit function, with different functions corresponding to different surface types. For the Gyroid structure studied here, the surface equation is as follows:

$$U_{G}\left(x,y,z\right)=\cos\left(x\right)\sin\left(y\right)+\cos\left(y\right)\sin\left(z\right)+\cos\left(z\right)\sin\left(x\right)-C$$ (1)

where *C* is a parameter that regulates the shape of the surface.

The porous architecture of the TPMS structures can be modulated by varying the implicit function, as the surface morphology is dictated by the expression. The surface is defined by Equation 1 serves as the interface between the solid and pore regions within a unit cell, where *U*_*G*_ > *C* corresponds to the solid phase, and regions where *U*_*G*_ < *C* correspond to the pore phase. By adjusting the threshold *C*, the geometry of the solid-pore interface can be altered, thereby changing the volume fractions of the two phases and consequently the porosity of the unit cell. Porosity *P* is defined as the volume fraction of pores in the total volume and is calculated as:

$$P=\left(\frac{V_{all}-V_{entity}}{V_{all}}\right)$$ (2)

where *V*_*all*_ is the total volume and *V*_*entity*_ is the volume of the solid TPMS structure.

By varying the threshold *C* in Equation 1, unit cells with different porosities can be generated. A linear relationship between the porosity *P* of the Gyroid cell and the threshold *C* was established through fitting:

P=kC+b (3)

where *k* and *b* are fitting constants.

Uniform porous structures were fabricated by spatially repeating a single unit cell type in a 4 × 4 × 4 array. All unit cells within a given uniform structure were designed with identical porosities (70%) and unit cell sizes. Specimens were labelled according to the overall array size, which was calculated as unit cell size × 4: Cube structures as C6, C10, C12; Octa structures as O6, O10, O12; and Gyroid structures as G6, G10, G12.

**FIGURE 1:**
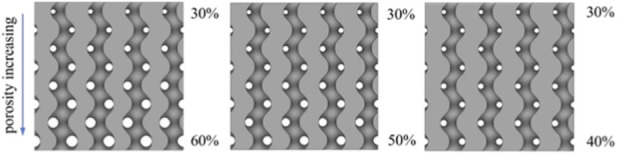
Cross sections with three gradient variation ranges.

For gradient porous structures, the porosity was varied linearly along one axis to prevent an excessive reduction in the stiffness associated with uniformly large pores. Three gradient ranges were designed, with a minimum porosity of 30% to maximum values of 60%, 50%, and 40%. These configurations are designated as G30-60, G30-50, and G30-40, respectively. Representative cross-sections illustrating these gradients are presented in Figure 1. The local pore size generated by each architecture is topology-dependent; therefore, the unit cell size was used as a controlled design variable rather than being treated as equivalent to the actual pore diameter.

### 2.2 Additive manufacturing of porous structures

Upon completion of the digital design, all models were exported in STL format and processed using Materialise Magics 21.0 (Materialise NV, Belgium) for repair and support generation. Block supports were strategically added to ensure structural integrity during manufacturing and to facilitate post-processing. The supported models were then fabricated using Selective Laser Melting (SLM) (Cao et al., 2021; Saremian et al., 2021). All specimens were fabricated using a YLM150 SLM system (Yongnian Laser, China) with Ti-6Al-4V titanium alloy powder with a particle size of 15–53 μm. The processing parameters were as follows: laser power = 140 W, scan speed = 1000 mm/s, layer thickness = 0.02 mm, hatch spacing = 0.05 mm, and laser frequency = 33 kHz. The build chamber was filled with argon gas to maintain the oxygen content below 0.1% during printing.

A total of 108 specimens were fabricated in this study, including 54 uniform porous structures (3 unit cell types × 3 unit cell sizes × 6 replicates) and 54 gradient porous structures (3 gradient ranges × 3 unit cell sizes × 6 replicates).

**FIGURE 2:**
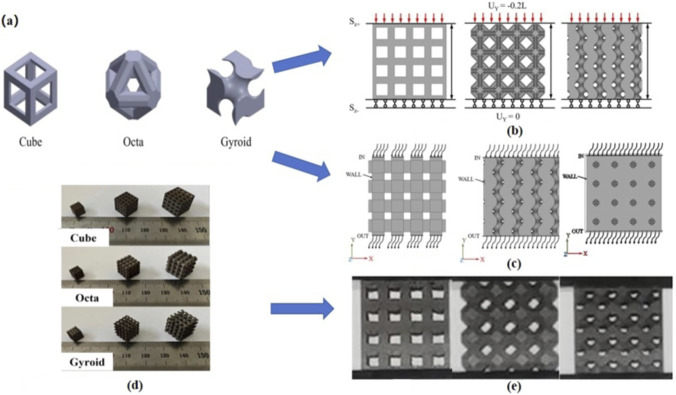
Schematic diagram of the workflow for characterizing the mechanical and permeability properties of uniform porous structures: **(a)** Types of unit cells used for uniform structures; **(b)** Mechanical simulation for mechanical property evaluation; **(c)** Fluid simulation for permeability calculation; **(d)** SLM-manufactured uniform porous structures, and **(e)** mechanical compression testing.

After printing, the specimens were removed from the build plate using wire electrical discharge machining (WEDM). The block supports were then manually removed using cutting pliers. Subsequently, all specimens were subjected to sandblasting to remove loosely attached powder particles and obtain a more consistent surface condition. Finally, a stress relief annealing treatment was performed at 600 °C for 2 h in a vacuum furnace, followed by furnace cooling, to reduce residual stresses induced during the SLM process. A schematic overview of the characterization workflow, including the manufactured uniform structures, is presented in Figure 2.

### 2.3 Compression test

The mechanical properties of the fabricated porous structures were evaluated under uniaxial compression using a KYZW50 testing machine. Each specimen was positioned centrally on the base plate. The upper platen was carefully aligned to ensure initial contact and proper calibration of the specimen. A constant crosshead displacement rate of 0.5 mm/min was applied under computer control until the structural failure was observed. Force-displacement data were recorded in real time and subsequently converted into engineering stress-strain curves for analysis. For each experimental condition, six replicate specimens were tested to ensure statistical reliability. All reported mechanical property values represent the mean ± standard deviation of the six replicates. The elastic modulus was calculated from the slope of the linear elastic region of the stress–strain curve within the strain range of 0.005–0.02. Statistical analysis was performed using one-way ANOVA followed by Tukey’s *post hoc* test, and p < 0.05 was considered statistically significant.

### 2.4 Numerical simulation

#### 2.4.1 Finite element modeling of porous structures

Finite element models were developed for one TPMS-based structure (Gyroid) and two conventional structures (Cube and Octa). The material was defined as a Ti-6Al-4V titanium alloy in all the simulations. The geometric models were imported into ABAQUS (v.2022, Dassault Systèmes, Paris) for mechanical analyses. Unit cell models with dimensions of 1.5 × 1.5 × 1.5 mm^3^, 2.5 × 2.5 × 2.5 mm^3^, and 3 × 3 × 3 mm^3^ were created in ABAQUS by specifying the strut dimensions.

For the permeability analysis, the unit cell models were assembled into 4 × 4 × 4 arrays using COMSOL Multiphysics (v.6.0, COMSOL AB, Sweden). A 4 × 4 × 4 configuration was selected to balance the computational efficiency and boundary condition effects. A sensitivity analysis confirmed that the relative difference in permeability between the 4 × 4 × 4 and 5 × 5 × 5 models was negligible (approximately 0.1%), indicating minimized boundary effects.

#### 2.4.2 Mechanical properties analysis

The compressive modulus and strength were evaluated to characterise the mechanical behaviour of the structures. The base material was set as Ti-6Al-4V with a Young’s modulus of 110.0 GPa and Poisson’s ratio of 0.34. The mechanical simulations were performed using ABAQUS/Explicit to account for the nonlinear deformation and possible contact behavior during compression. Ti-6Al-4V was assigned as the base material for the uniform porous structures. In the elastic stage, the Young’s modulus and Poisson’s ratio were set as 110 GPa and 0.34, respectively. Plastic deformation of Ti-6Al-4V was described using a Johnson-Cook constitutive model according to previously reported material parameters. In the present FE model, plastic deformation was included, whereas no explicit damage evolution, fracture criterion, or element deletion-based failure model was implemented. Therefore, the FE simulation was mainly used to reproduce the elastic-plastic compressive response and estimate the equivalent elastic modulus and compressive strength, while the final fracture/failure behavior was identified from the experimental compression stress-strain curves. Geometric nonlinearity was enabled in the compression simulations. A vertical displacement of 0.2 L was applied to the top surface, where *L* is the initial height of the specimen, while the bottom surface was constrained in the compression direction and allowed to deform freely in the lateral directions. Mesh convergence was checked by comparing the predicted elastic modulus and compressive strength under different mesh densities, and the final mesh was selected when further mesh refinement produced negligible changes in the calculated mechanical response. The boundary conditions applied to the FE model were as follows: a vertical displacement load of 0.2 L was applied to the top surface (*S*_*z*+_) along the *z*-axis; the bottom surface (*S*_*z*-_) was fixed in the *z*-direction while allowing free deformations in the *x* and *y* directions. The boundary conditions for the three porous structures are shown in Figure 2c. Quasi-static compression conditions were used to simulate the mechanical response. The porous structures were meshed using C3D8R eight-node linear hexahedral elements with reduced integration. The average mesh size was set to 0.1 mm, and mesh convergence analysis showed that reducing the mesh size below 0.12 mm resulted in a relative change of less than 2% in the predicted mechanical response. Self-contact was defined with a friction coefficient of 0.1 to account for possible contact between internal struts during compression.

#### 2.4.3 Permeability analysis

All porous structures selected for the permeability analysis had a porosity of 70% ± 0.1%. The STL files of the structures were imported into Materialise Magics for geometric refinement, which included removing intersecting or overlapping facets and patching surface holes to ensure the mesh quality. A fluid flow domain was created by generating a solid cube with the same outer dimensions as the porous structure in Magics and performing a Boolean subtraction operation.

The resulting fluid domain model was imported into COMSOL for the CFD simulation. Assuming a low flow velocity and small characteristic dimensions, the inertia term in the Navier-Stokes equations was neglected, and a steady-state laminar flow model was adopted. The fluid was defined as cell culture medium, with a density ρ = 1000 kg/m^3^ and dynamic viscosity μ = 1 × 10^−3^ Pa·s. The Reynolds number was calculated using *Re = ρvd/μ*, where *ρ* = 1000 kg/m^3^ is the density of the fluid medium, *v* = 1 × 10^−4^ m/s is the inlet velocity, *d* = 0.5 mm is the characteristic pore size, and *μ* = 1 × 10^−3^ Pa·s is the dynamic viscosity. The permeability was calculated according to Darcy’s law:

$$k=\frac{\mu QL}{A\Delta P}=\frac{\mu v_{s}L}{\Delta P}$$ (4)

where *K* is the permeability, *μ* is the dynamic viscosity of the fluid, *v* is the superficial inlet velocity, *L* is the length of the flow domain along the flow direction, and *ΔP* is the pressure drop between the inlet and outlet surfaces. The prescribed inlet velocity was 1.0 × 10^−4^ m/s, and the outlet pressure was set to zero gauge pressure. The velocity used in the permeability calculation refers to the superficial velocity applied at the inlet boundary rather than the local pore velocity. The calculated Reynolds number is approximately 0.05, indicating that the flow remains within a low-Reynolds-number laminar regime. Therefore, inertial effects are negligible, and the use of the Darcy flow assumption is reasonable for the present permeability simulations. The boundary conditions were set as follows: a velocity inlet (*v* = 1 × 10^−4^ m/s) was applied at the inflow surface, zero gauge pressure was set at the outlet, and no-slip conditions were imposed on all solid walls. For the simulations along a single axis, the surfaces perpendicular to that axis were assigned as the velocity inlet and pressure outlet, respectively, with the remaining surfaces treated as walls.

In the COMSOL setup, the y-axis was selected as the flow direction for the uniform porous structures. The *S*_*Y+*_ surface was defined as the velocity inlet, where a superficial velocity of 1.0 × 10^−4^ m/s was applied. The *S*_*Y−*_ surface was defined as the pressure outlet, where the gauge pressure was set to 0 Pa. All remaining surfaces were treated as no-slip wall boundaries.

## 3 Results

### 3.1 Size effects on the mechanical properties of structures

#### 3.1.1 Size effect on the stress distribution

**FIGURE 3:**
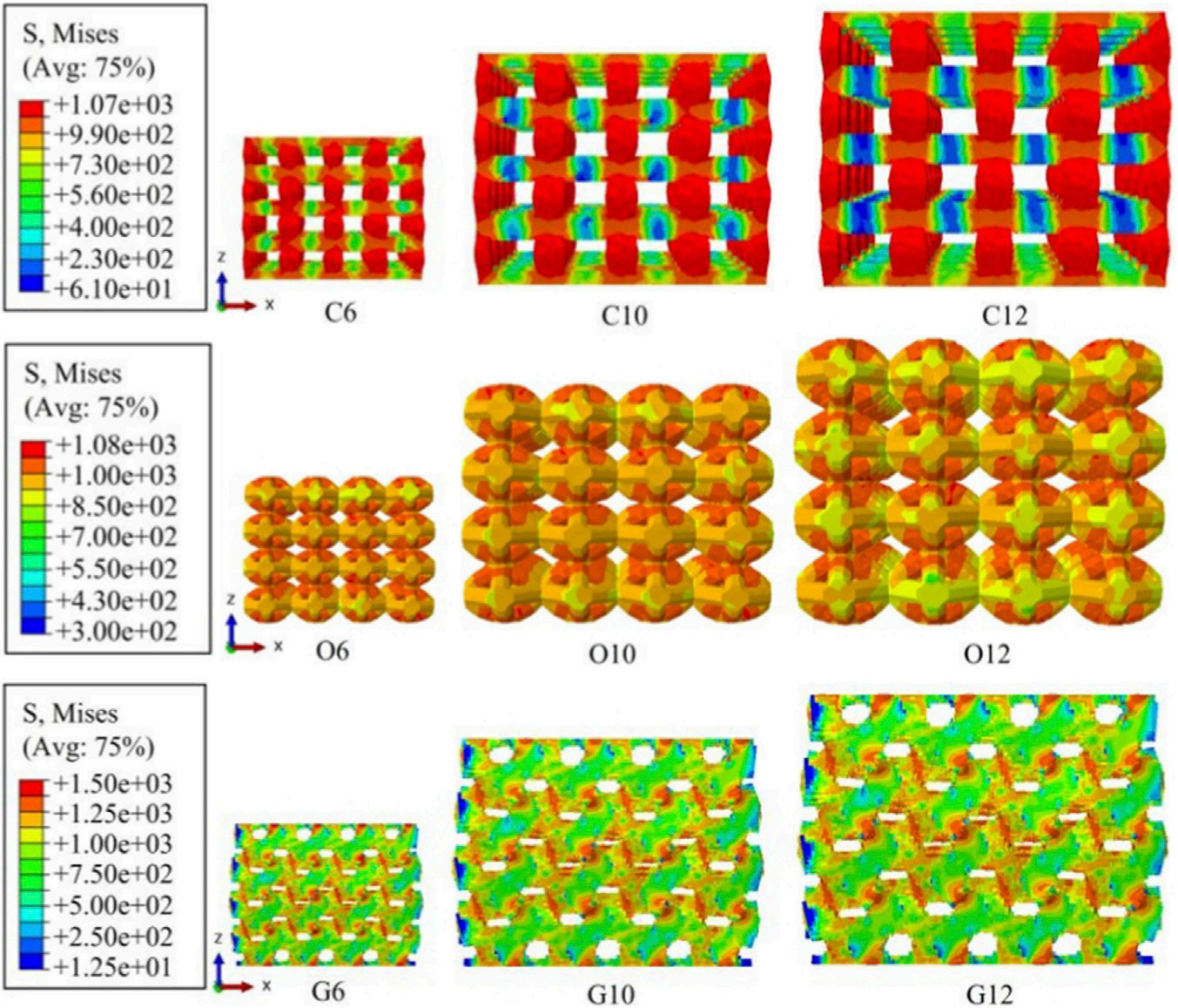
Von Mises stress distributions in uniform porous structures with different unit cell sizes under uniaxial compression.

The stress distributions for the three uniform porous structures with different unit cell sizes under compressive loading are illustrated in Figure 3. Distinct stress concentration patterns were observed, depending on the geometry. In the Gyroid structure, stress is concentrated at the pore junctions and curved regions.

Stress concentration was primarily observed along the axial struts of the Cube structure, with the maximum value occurring at the strut midspans. For the Octa structure, the stress was localised at the prismatic junctions between the cells. Although a more uniform stress distribution was achieved for the Octa structure than the Cube structure, a higher overall stress level was exhibited. It is generally observed that a more diffuse and uniform stress distribution is achieved by reducing the unit cell size, which is particularly beneficial for the Gyroid structure.

**FIGURE 4:**
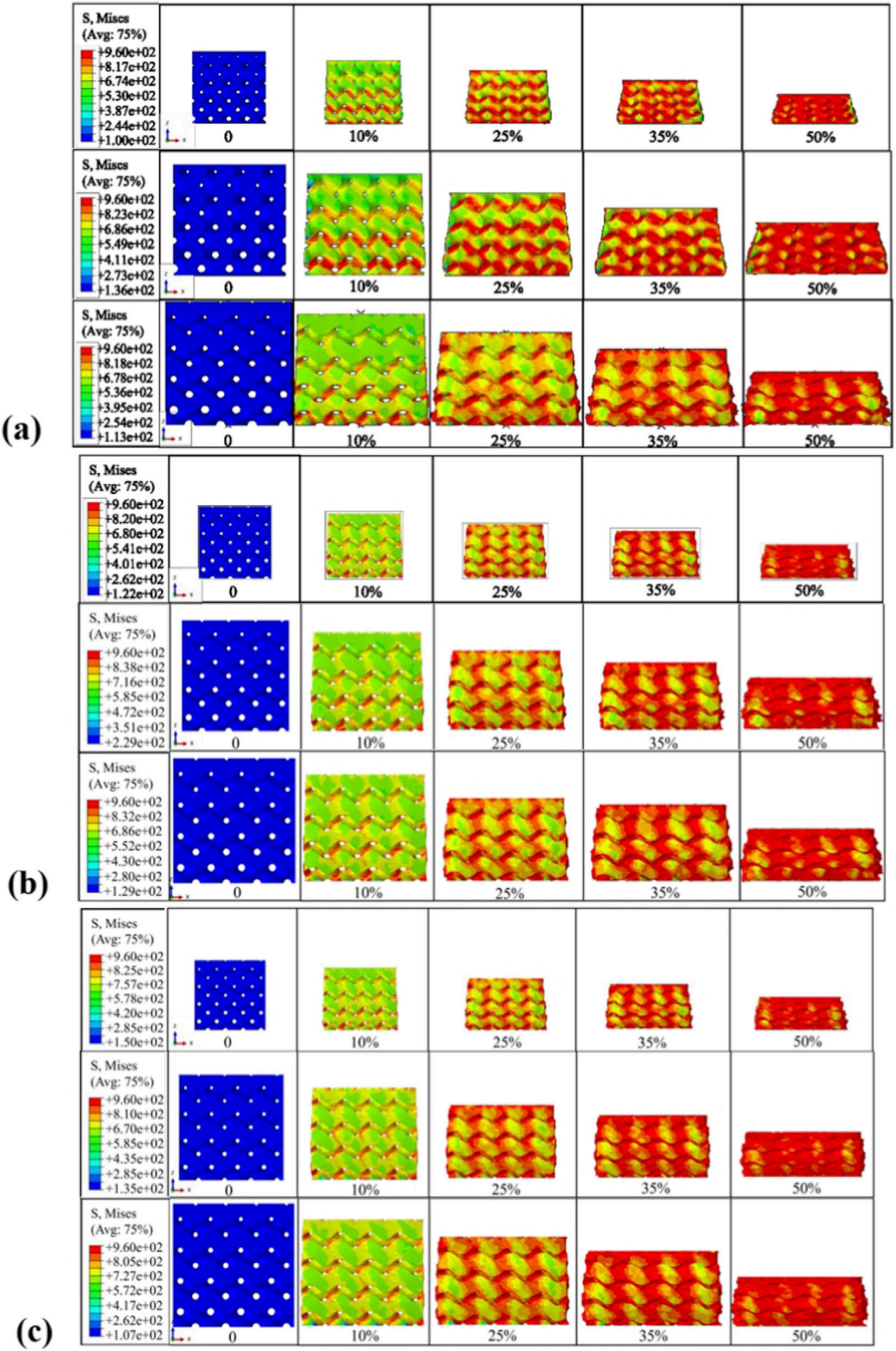
Stress distributions in the gradient porous structures during the compression process: **(a)** Stresses in G30-60 structures, **(b)** Stresses in G30-50 structures, **(c)** Stresses in G30-40 structures.

The von Mises stress distributions of the gradient porous structures under compressive loading are shown in Figure 4, with a generally non-uniform distribution across the entire structure. At an engineering strain of approximately 10%, significant stress concentrations were detected in the high-porosity regions, particularly at the curved transition regions connecting adjacent pores with different dimensions. As the strain increased, the structural collapse initiated in the low-porosity regions with higher stiffness, and progressively propagated towards the high-porosity zones.

A detailed comparison reveals that as the gradient range narrows (e.g., from G30-60 to G30-40), the stress state increasingly resembles that of a uniform porous structure. For the widest gradient (G30-60), the influence of the unit cell size on the average stress was more pronounced, diminishing as the gradient range decreased. The stress concentration was more pronounced in smaller unit cell models (e.g., 6.0 mm) than that in larger ones (e.g., 12.0 mm).

#### 3.1.2 Size effect on the equivalent compression modulus and strength

Mechanical testing confirmed that all three structures (Cube, Octa, and Gyroid) underwent elastic deformation during compression. A sharp stress drop accompanied the fracture of the porous structures, which was attributable to the inherent brittleness of the Ti-6Al-4V alloy, in contrast to the ductile response typical of 316 L stainless steel.

**FIGURE 5:**
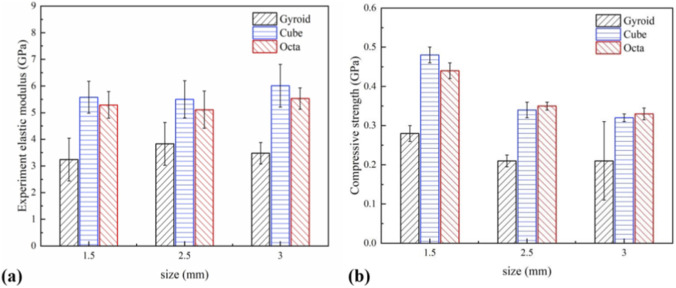
Comparison of mechanical properties for uniform porous structures: **(a)** relationship between unit cell size and experimental elastic modulus, and **(b)** relationship between unit cell size and compressive strength. Data are presented as mean ± standard deviation (n = 6).

The compressive moduli and strengths of the uniform structures are compared in Figure 5. Compared to the compressive strength, the compressive modulus was less sensitive to changes in the unit cell size. For example, the modulus of the Cube structure varied within a narrow range of approximately 5.5 GPa (Figure 5a). In contrast, the compressive strength of all three structures decreased significantly with increasing unit cell size, particularly between 1.5 and 2.5 mm (Figure 5b). The Cube and Octa structures exhibited higher compressive strength than the Gyroid structure, with the Octa structure showing comparable or slightly higher strength in most size groups. Notably, the Gyroid structure exhibited the highest fracture strain, suggesting better deformation tolerance.

**FIGURE 6:**
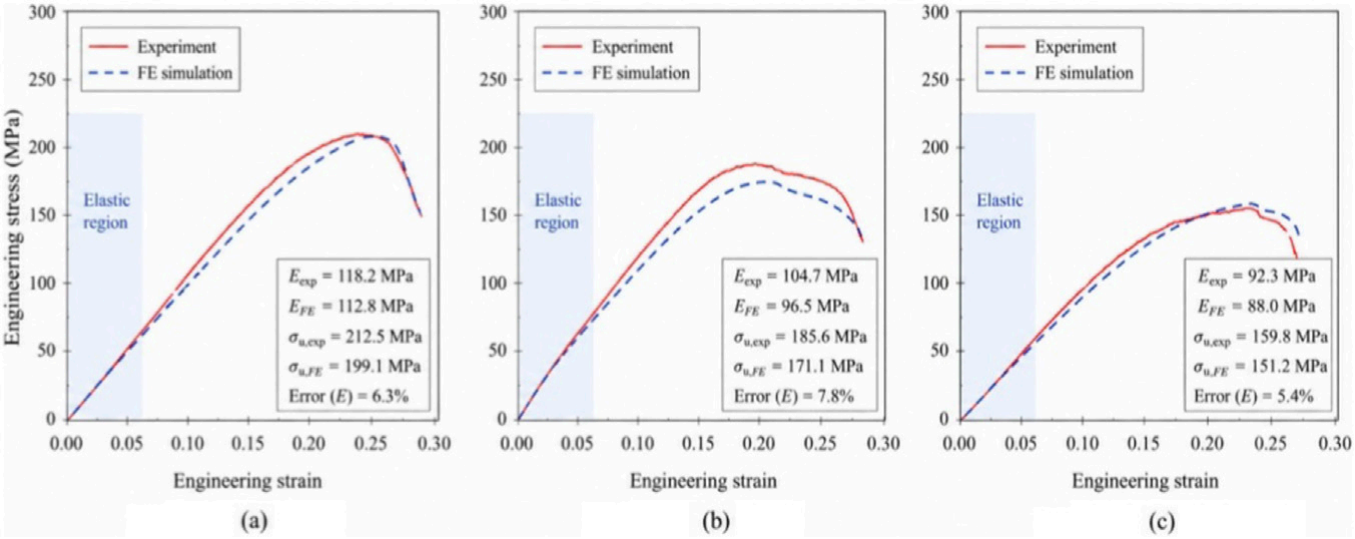
Comparison between experimental and FE simulated compressive stress–strain curves for representative porous structures with a unit cell size of 2.5 mm. **(a)** Cube; **(b)** Octa; **(c)** Gyroid.

To verify the reliability of the finite element model, the simulated compressive stress–strain curves were compared with the experimental results for representative porous structures with a unit cell size of 2.5 mm, as shown in Figure 6. The FE-simulated curves exhibited good agreement with the experimental results in terms of elastic response, compressive strength, and overall deformation behavior. The relative errors of compressive strength between the FE simulation and experimental results were 6.3%, 7.8%, and 5.4% for the Cube, Octa, and Gyroid structures, respectively. These results indicate that the developed numerical model can reasonably predict the mechanical response of porous structures under compression loading. The decrease in strength with increasing cell size at constant porosity (70%) is attributed to the fact that while the wall thickness increases, the concurrent increase in pore size (and thus strut length) has a more dominant weakening effect, making the structure more susceptible to buckling.

A quantitative summary of the mechanical and permeability properties of the nine uniform porous structures is provided in Table 2. Overall, the Cube and Octa structures exhibited higher elastic modulus and compressive strength than the Gyroid structure, whereas the Gyroid structure showed higher fracture strain and better deformation tolerance. With increasing unit cell size, the compressive strength of all three structures decreased, while permeability increased markedly. This indicates a trade-off between load-bearing capacity and fluid transport performance under the same target porosity.

**TABLE 2:** Quantitative summary of mechanical and permeability properties of uniform porous structures.

Structure	Unit cell size (mm)	Elastic modulus (GPa)	Compressive strength (GPa)	Fracture strain (%)	Permeability (×10^–9^ m^2^)
Cube	1.5	5.5 ± 0.6	0.48 ± 0.02	14.0 ± 0.8	17.3
Cube	2.5	5.5 ± 0.7	0.34 ± 0.02	11.0 ± 0.6	47.4
Cube	3.0	6.0 ± 0.8	0.32 ± 0.02	10.5 ± 0.5	67.6
Octa	1.5	5.3 ± 0.5	0.44 ± 0.02	17.0 ± 0.9	8.7
Octa	2.5	5.2 ± 0.6	0.35 ± 0.01	14.0 ± 0.7	24.0
Octa	3.0	5.5 ± 0.4	0.33 ± 0.02	12.0 ± 0.6	25.4
Gyroid	1.5	3.2 ± 0.8	0.28 ± 0.02	20.5 ± 1.0	13.1
Gyroid	2.5	3.9 ± 0.8	0.21 ± 0.02	14.5 ± 0.8	35.7
Gyroid	3.0	3.5 ± 0.4	0.21 ± 0.10	12.5 ± 0.7	51.6

Elastic modulus, compressive strength, and fracture strain values are presented as mean ± standard deviation (n = 6). Fracture strain was determined from the experimental stress–strain curves. Permeability values were obtained from CFD simulations of the designed porous structures and are reported in units of ×10^–9^ m^2^. Since permeability was not measured from repeated physical tests or independently reconstructed numerical specimens, no standard deviation or statistical significance analysis is reported for permeability.

**FIGURE 7:**
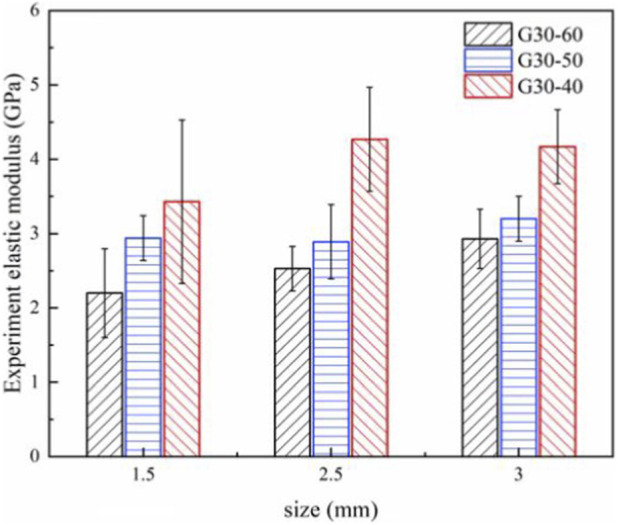
Comparison of mechanical properties for gradient porous structures.

For the gradient porous structures (Figure 7), larger cell sizes corresponded to higher stress levels at identical strains during the elastic stage. This size-dependent stress gap was most evident in the G30-60 structure and diminished as the gradient range narrowed. This indicates that the compressive response of gradient structures is more sensitive to cell size when the porosity variation is significant. The elastic modulus was the highest for G30-40 and the lowest for G30-60, reflecting their different average porosities. The modulus increased with the cell size, most notably between 1.5 and 2.5 mm.

### 3.2 Size effects on the structural permeability properties

#### 3.2.1 Analysis of pressure drop within the structure

**FIGURE 8:**
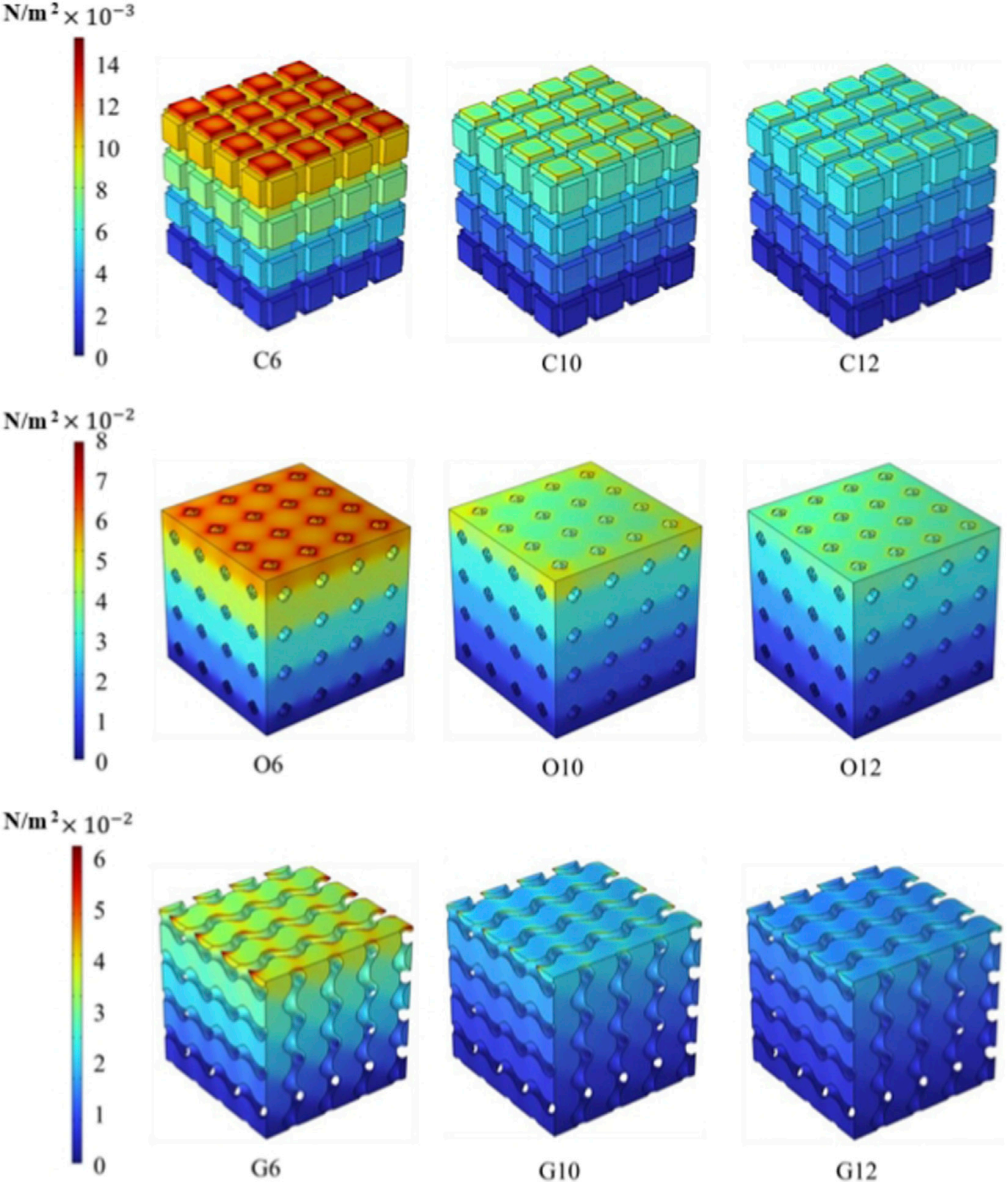
Pressure drops in uniform porous structures.

Figure 8 shows the simulated pressure distributions for the three uniform structures tested. The pressure was highest at the inlet and decayed to zero at the outlet, which was consistent with the imposed boundary conditions. When comparing structures with the same porosity, the Octa model, which has its more complex flow path, produced the highest pressure drop. The Cube model, with simpler straight channels, showed the lowest pressure drop. The Gyroid structure exhibited an intermediate pressure drop, with the inlet pressure concentrated at the curved junctions.

When comparing different unit cell sizes for the same structure type, the pressure drop decreased significantly as the unit cell size increased. The reduction was most pronounced between the 1.5 mm and 2.5 mm unit cell groups. The difference in the pressure drop between 2.5 and 3.0 mm cell sizes was less pronounced.

**FIGURE 9:**
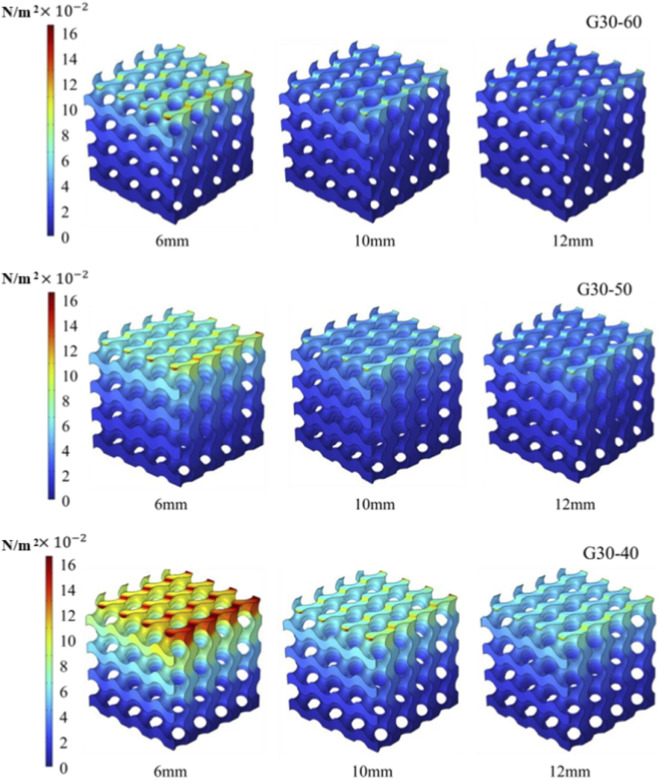
Pressure drops in gradient porous structures.

The pressure distributions for the gradient structures are shown in Figure 9 and summarised in Table 3. The G30-40 structure (smallest gradient range/highest average density) resulted in the highest pressure drop, whereas G30-60 (largest gradient range/lowest average density) exhibited the lowest pressure drop. This confirms that the average porosity is the dominant factor governing permeability. When the fluid flows perpendicular to the gradient direction, the behaviour approximates that of a uniform structure with local porosity. For a given gradient range, smaller cell sizes (6 mm) consistently led to higher pressure drops, which is consistent with the conclusions drawn from the uniform structures. The magnitude of the pressure drop variation decreased with the increasing cell size.

**TABLE 3:** Maximum pressure drop for different gradient structures.

Maximum pressure drop (Pa)	Overall array size (mm) 6 mm	Overall array size (mm) 10 mm	Overall array size (mm) 12 mm
G30-60	11.60	4.53	3.05
G30-50	13.77	5.27	3.81
G30-40	19.00	6.77	4.64

#### 3.2.2 Analysis of structural permeability

**FIGURE 10:**
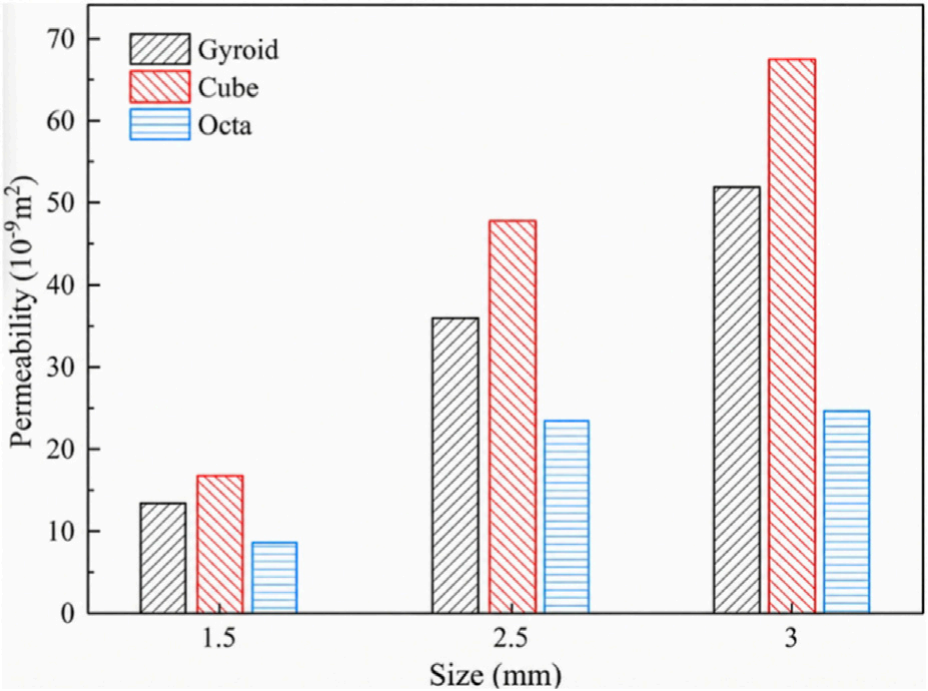
Comparison of CFD-derived permeability values in uniform porous structures.

The permeability values for all uniform structures fell within the range of 10^–9^ to 10^–8^ m^2^ (Figure 10). A clear trend was observed: permeability increased with the increase of unit cell size for all three structure types. Importantly, the disparity in permeability between the different structures became more pronounced at larger scales. For a 1.5 mm cell, the permeability of the Octa structure was only approximately 4.9% lower than that of the Cube structure. However, for a 3.0 mm cell, the permeability of Octa was 62.7% lower than that of Cube, indicating that the geometric effects on fluid flow were amplified in larger cells. The permeability values were obtained from CFD simulations of the designed porous structures. Since these values were not obtained from repeated physical permeability tests or independently reconstructed numerical specimens, statistical error bars and significance analysis were not applied to the permeability results. Since the permeability values were obtained from CFD simulations rather than repeated physical experiments, conventional statistical significance analysis was not performed. For a 1.5 mm cell, the permeability of the Octa structure was only approximately 4.9% lower than that of the Cube structure. However, for a 3.0 mm cell, the permeability of Octa was 62.7% lower than that of Cube, indicating that the geometric effects on fluid flow were amplified in larger cells. The permeability values were obtained from CFD simulations of the designed porous structures. Since these values were not obtained from repeated physical permeability tests or independently reconstructed numerical specimens, statistical error bars and significance analysis were not applied to the permeability results.

**FIGURE 11:**
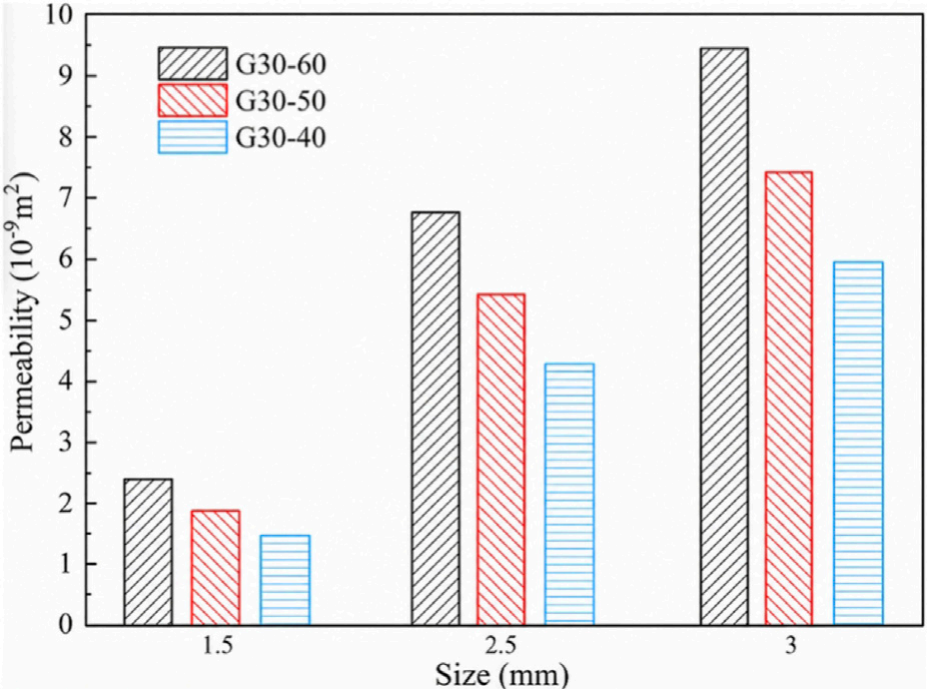
Comparison of CFD-derived permeability in different gradient porous structures.

For the gradient structures (Figure 11), the permeability increased with both the gradient range (i.e., average porosity) and the unit cell size. Structures with the same gradient range showed increased permeability with larger cell sizes. The rate of this increase was more pronounced for structures with higher average porosities (e.g., G30-60). For instance, the permeability of the 12.0 mm G30-60 structure was approximately 300% higher than that of its 6.0 mm counterpart, a trend similar to that observed for the uniform Gyroid structure.

#### 3.2.3 Comparison with theoretical permeability models

**FIGURE 12:**
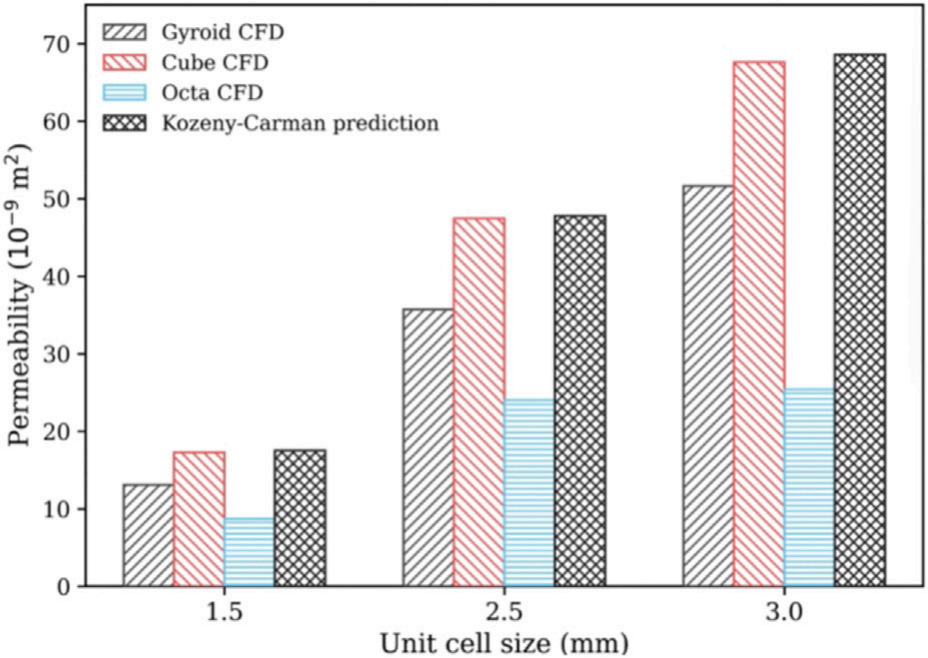
Comparison between CFD-derived permeability values and theoretical predictions based on the Kozeny-Carman relationship for different unit-cell topologies.

To further examine whether the size-dependent permeability trend obtained from the CFD simulations is consistent with classical permeability theory, the CFD-derived permeability values were compared with theoretical predictions based on the Kozeny-Carman relationship. The Kozeny-Carman model provides a theoretical baseline for porous media by relating permeability to porosity and characteristic pore size. The theoretical permeability was calculated using the following simplified Kozeny-Carman equation

$$k_{kc}=\frac{\varepsilon^{3}d_{eff}^{2}}{C{\left(1-\varepsilon \right)}^{2}}$$ (5)

where *k*_*KC*_ is the theoretical permeability, ε is the porosity, *d*_*eff*_ is the effective characteristic pore size, and *C* is the Kozeny constant. In this study, *ε* = 0.70, *d*_*eff*_ = 0.6a, where a is the unit cell size, and *C* = 180. This calculation was used as a theoretical baseline for comparison with the CFD-derived permeability values rather than an exact representation of the complex internal flow channels. As shown in Figure 12, the Kozeny-Carman prediction captured the overall increasing trend of permeability with increasing unit cell size. The Cube structure exhibited the closest agreement with the theoretical prediction, with relative deviations below 2%. The Gyroid structure showed a moderate deviation of approximately 24%–25%, which may be attributed to its curved and continuous flow channels. In contrast, the Octa structure showed a larger deviation, especially at larger unit cell sizes, probably due to its more complex internal geometry and local flow constriction effects. These results indicate that although the overall size-dependent permeability trend is consistent with classical permeability theory, the absolute permeability values are strongly affected by unit-cell topology.

## 4 Discussion

In this study, we systematically investigated the influence of unit cell size (the size effect) on the mechanical properties and permeability of both uniform and gradient porous lattices, through an integrated experimental and numerical approach including SLM fabrication, uniaxial compression testing, FE numerical simulation, and CFD simulation. Our findings provide quantitative design rules for lightweight components in aerospace, automotive, and other engineering fields, as well as design references for porous scaffolds where mechanical integrity and fluid transport need to be balanced. The experimental and numerical results showed generally consistent trends in the mechanical response of the investigated porous structures. The FE-predicted stress–strain curves agreed well with the experimental curves in the elastic and early plastic deformation stages. The relative errors of compressive strength between the FE simulation and experimental results were 6.3%, 7.8%, and 5.4% for the Cube, Octa, and Gyroid structures, respectively. These results suggest that the developed FE model can provide reasonable estimates of the equivalent elastic modulus and compressive strength of regular porous structures under compression, particularly in the elastic and early plastic deformation stages. The remaining deviations may be attributed to manufacturing-induced imperfections, including surface roughness, partially adhered powder particles, local dimensional deviations, and possible micro-defects generated during SLM fabrication, which were not explicitly considered in the idealized CAD-based FE models. Nevertheless, the FE model should be interpreted as a tool for reproducing the elastic–plastic compressive response and comparing relative trends rather than as a complete fracture model. Since no explicit damage evolution, fracture criterion, or element deletion scheme was implemented, the final collapse and fracture behavior were primarily identified from the experimental stress–strain curves. Therefore, the numerical results should be interpreted primarily for comparing relative trends among different unit-cell sizes and topologies, while experimentally measured fracture strain and post-peak response remain necessary for evaluating structural failure. The conclusions of the present study should therefore be interpreted as design references for controllable periodic porous structures, while further work is needed to verify whether similar size-dependent trends exist in irregular or hybrid scaffold architectures.

Currently, the design of bone tissue engineering scaffolds still faces a long-standing challenge: balancing sufficient mechanical strength to match native bone tissue and adequate permeability to support nutrient transport and cell ingrowth. While extensive studies have confirmed that porosity and unit cell topology are key determinants of scaffold performance, systematic investigations focusing on the isolated effect of unit cell size, independent of porosity, remain relatively scarce. In this work, we fixed the overall porosity at 70% to eliminate the interference of porosity variation, and clearly revealed that unit cell size acts as an independent regulatory factor for both mechanical and fluid transport properties of porous structures. Our compression test results showed that reducing the unit cell size effectively mitigated stress concentrations, especially for the TPMS-based Gyroid structure, which exhibited the most uniform stress distribution among the three architectures. This is attributed to the zero mean curvature characteristic of Gyroid surfaces, which enables smoother stress flow, and the denser spatial arrangement of smaller unit cells further disperses the stress at the structural junctions. Meanwhile, we found that the compressive strength of all three structures decreased significantly with increasing unit cell size, particularly in the range of 1.5 mm–2.5 mm, while the elastic modulus showed a relatively low sensitivity to size variation. This size-dependent mechanical behavior is mainly caused by the fact that the increase in strut length and pore size with larger unit cells has a more dominant weakening effect than the increase in wall thickness, making the structure more prone to buckling and fracture.

From the perspective of fluid transport performance, our CFD simulation results demonstrated a significant positive correlation between permeability and unit cell size for all structures. A pronounced performance transition was observed between the 1.5 mm and 2.5 mm unit cell groups. Compared with the 2.5 mm and 3.0 mm groups, the 1.5 mm structures exhibited substantially higher flow resistance and pressure drop, indicating that reducing the unit cell size within this range markedly restricts the flow channels. Since no 2.0 mm group was included in the present study, this transition should be interpreted as an observed interval between 1.5 and 2.5 mm rather than a precise critical threshold. Compared with traditional strut-based Cube and Octa structures, the Gyroid structure exhibited a more balanced performance profile. Although the Octa structure showed higher compressive strength and the Cube structure exhibited higher elastic modulus, the Gyroid structure demonstrated a more uniform stress distribution, higher fracture strain, and a favorable balance between mechanical response and permeability. These characteristics suggest that the Gyroid architecture may serve as a promising candidate for further scaffold design.

Several limitations should be acknowledged. First, the present study focused on three representative regular architectures, namely Cube, Octa, and Gyroid, and did not include irregular Voronoi or Laguerre**–**Voronoi porous structures. Therefore, the conclusions are most directly applicable to periodic porous structures with controllable unit-cell size and porosity, rather than to stochastic trabecular-like scaffolds. Second, the uniform structures were designed at a fixed target porosity of 70%, and the selected unit-cell sizes were limited to 1.5 mm, 2.5 mm, and 3.0 mm. Although this controlled design enables the isolated effect of unit-cell size to be evaluated, a broader parametric space involving different porosity levels, pore-size distributions, and hybrid topologies should be explored in future work. Third, the permeability values were obtained from CFD simulations based on idealized designed geometries, rather than from direct permeability experiments or μCT-reconstructed as-built geometries. Therefore, the permeability results should be interpreted mainly as comparative trends among different architectures and unit-cell sizes. Future studies should combine experimental permeability measurements and image-based numerical reconstruction to further validate the predicted fluid-transport behavior. Finally, the present work evaluated quasi-static compressive behavior, while fatigue, torsion, shear, biological response, and long-term *in vivo* performance were not considered. Therefore, the results should be interpreted as structural design references rather than direct evidence supporting clinical application.

## 5 Conclusion

In summary, this study systematically investigated the influence of unit cell size (the size effect) on the mechanical properties and permeability of porous lattices using integrated experimental and numerical approaches. Rather than proposing a new porous topology, this work provides a controlled comparison of representative regular porous architectures and clarifies how unit-cell size affects the balance between compressive performance and permeability under fixed or prescribed porosity conditions. The main conclusions are as follows:

1. Among the three porous architectures, the Cube structure exhibited a relatively high elastic modulus, whereas the Octa structure showed the highest compressive strength. The Gyroid structure demonstrated the most uniform stress distribution and the highest fracture strain, indicating better deformation tolerance. Therefore, rather than exhibiting the “best” mechanical properties in a single indicator, the Gyroid structure showed the most balanced mechanical response among the three architectures. In addition, reducing the unit cell size effectively mitigated stress concentration and improved structural stability, while the size effect was more pronounced within the transition interval between 1.5 mm and 2.5 mm.
2. For gradient porous structures, both the unit cell size and porosity gradient range significantly influence the mechanical and permeability properties. A narrower gradient range results in higher stiffness and lower permeability, making it more suitable for load-bearing scaffold regions, whereas a wider gradient range enhances permeability with moderate strength and may be beneficial for regions requiring improved nutrient transport.
3. The permeability of all three types of uniform porous structures was in the range from 1.0 × 10^−9^ to 1.0 × 10^−8^ m^2^. For both uniform and gradient porous structures, permeability increased with increasing unit cell size, showing an approximately linear relationship. The magnitude of this size-dependent effect was influenced by the unit cell topology and porosity gradient range.

## Data Availability

The original contributions presented in the study are included in the article/supplementary material, further inquiries can be directed to the corresponding author.
